# Treatment delay and tumor size in patients with oral cancer during the first year of the COVID‐19 pandemic

**DOI:** 10.1002/hed.26858

**Published:** 2021-09-08

**Authors:** Karl Metzger, Jan Mrosek, Sven Zittel, Maximilian Pilz, Thomas Held, Sebastian Adeberg, Oliver Ristow, Jürgen Hoffmann, Michael Engel, Christian Freudlsperger, Julius Moratin

**Affiliations:** ^1^ Department of Oral and Cranio‐Maxillofacial Surgery Heidelberg University Hospital Heidelberg Germany; ^2^ Institute of Medical Biometry and Informatics Heidelberg University Hospital Heidelberg Germany; ^3^ Department of Radiation Oncology Heidelberg University Hospital Heidelberg Germany

**Keywords:** COVID‐19, oral squamous cell carcinoma, pandemic, Sars‐CoV‐2, treatment delay

## Abstract

**Background:**

We set out to investigate how the ongoing coronavirus pandemic affected the size of tumors and the duration of treatment delay in patients with surgically treated oral squamous cell carcinoma.

**Methods:**

Patients with surgically treated oral cavity squamous cell carcinoma were assessed retrospectively and divided into two groups depending on when they had first presented at our clinic. Patients presenting from 2010 to 2019, that is, before COVID‐19 onset (*n* = 566) were compared to patients presenting in 2020 (*n* = 58).

**Results:**

A total of 624 patients were included. Treatment delay was significantly longer in 2020 (median = 45 days) versus 2010–2019 (median = 35 days) (*p* = 0.004). We observed a higher pathological T classification in 2020 (*p* = 0.046), whereas pathological N classification was unchanged between groups (*p* = 0.843).

**Conclusions:**

While extraordinary efforts continue to be made in the context of the pandemic, it is imperative that this does not lead to significant disadvantages for many people with oral cancer.

## 1 INTRODUCTION

By the end of December 2020, the coronavirus disease (COVID‐19), which is caused by severe acute respiratory syndrome coronavirus 2 (SARS‐CoV‐2), had affected more than 79 000 000 people worldwide.^1^ COVID‐19 has placed a severe burden not only on patients' mental and physical health, but also on health care resources. During the pandemic, surgical departments were forced to postpone surgeries due to an actual or anticipated shortage of critical care capacity. This has been particularly challenging for oncologists, who must consider potentially life‐threatening delays in cancer diagnosis and treatment and prioritize accordingly. The impact of delayed cancer treatment on survival rates varies substantially across cancer types and affects disproportionally patients with oral cavity cancers.^2^ At the same time, social distancing measures and fear of COVID‐19 can introduce additional burdens for patients and disrupt their care. Such additional delays may allow for significant tumor growth overall and put patients at an increased risk for adverse treatment outcomes. Therefore, international experts feared that treatment delays and increases in treatment duration caused by secondary effects of the pandemic would impact patients' morbidity and mortality.^3^ At our hospital, we experienced a severe shortage of critical care capacity during the pandemic, and we were interested in the extent to which treatments were affected. Therefore, we investigated whether treatment delay and tumor sizes were altered in patients with surgically treated oral squamous cell carcinoma, who presented during the pandemic in 2020, versus patients that had presented before 2020.

## 2 MATERIALS AND METHODS

After receiving approval by the Ethics Committee of Heidelberg University (Ethic vote: S‐183/2015) and the written informed consent by each patient, we retrospectively reviewed the medical records of patients with pathologically confirmed primary oral squamous cell carcinoma that had presented between January 1, 2010 and December 31, 2020. All procedures were in accordance with the Helsinki Declaration of 1975 (revised in 1983). All patients were treated surgically at the Department of Oral and Cranio‐Maxillofacial Surgery of the Heidelberg University Hospital according to German national guidelines.^4^ Patients with a history of malignancies prior to their tumor diagnosis were excluded from this protocol. Adjuvant treatment (radio‐ or radiochemotherapy) was performed in patients with relevant risk factors, that is, an advanced tumor size with cervical metastasis and/or positive histological risk factors. Patients without a curative treatment option were excluded from this protocol. After the medical records were reviewed, patients were divided into two groups depending on the date of their first presentation at our clinic. Group A included all patients presenting from 2010 to 2019, while patients presenting in 2020 were assigned to Group B. We decided to compare the entire calendar year of 2020 to the years prior to it, because (1) it is difficult to define an exact date for the onset of COVID‐19‐related anxiety in patients, which could have resulted in patient delays, and (2) shortages in medical care that may have led to extended treatment delays did not necessarily coincide precisely with government restrictions. We defined treatment delay as the number of days between a patient's first presentation at our clinic and the date of their surgery.

Clinical data and histopathological parameters were assessed using SAP Patient Management research software (SAP, Walldorf, Germany). We obtained information on patients' age, sex, treatment delay, pathologic T classification, pathologic N classification, and tumor stage according to Union for International Cancer Control (UICC) classification. We used Pearson's chi‐square test was used for comparisons of categorical data and Welch's unequal variances *t* test was used for comparisons of continuous data (*p*‐values below 0.05 were considered statistically significant). Statistical analyses were performed using the software R (version 4.0.3.).

## 3 RESULTS

**FIGURE 1:**
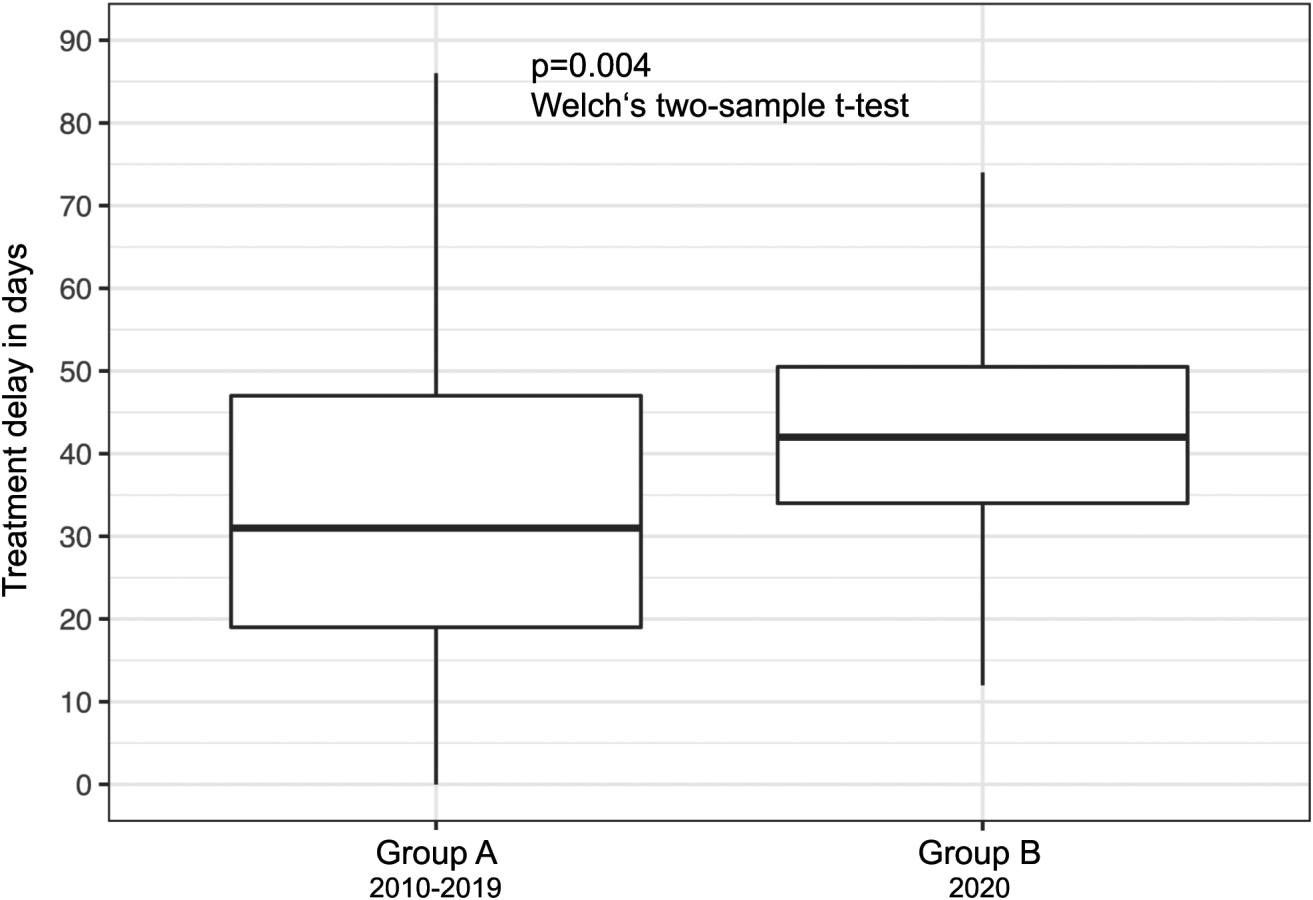
Comparison of treatment delay of group A versus group B. Treatment delay was significantly prolonged in group B, with a median delay of 45 days (Q1–Q3: 34–52 days), compared to a delay of 35 days (Q1–Q3: 19–47 days) in group A (*p* = 0.004; Welch's two‐sample *t* test)

A total of 624 patients matched our inclusion criteria. The relevant demographic and clinical characteristics of patients are displayed in Table 1. Of the included patients, 366 patients were male (59%) and 258 were female (41%). The overall median patient age at the time of initial diagnosis of oral squamous cell carcinoma was 65 years (Q1–Q3: 56–74 years) with a range of 18–95 years. We found tumors of following size classification across our cohort: Tx: 1 (1%), T1: 218 (35%), T2: 171 (27%), T3: 51 (8%), and T4: 183 (29%). According to the UICC classification, 287 patients (46%) presented with early‐stage disease (UICC I/II) and 337 patients (54%) presented with advanced‐stage disease (UICC III/IV). All patients that were scheduled for primary surgical therapy could be treated surgically and there were no COVID‐related changes in treatment modality. The overall median treatment delay was 34 days (Q1–Q3: 20–48 days). Group A (presenting in 2010–2019) contained 566 patients (i.e., on average 57 patients per year) and group B (presenting in 2020) contained 58 patients. Therefore, we did not observe a decrease in the incidence rate during 2020. Treatment delay was significantly longer in patients of group B, with a median delay of 45 days (Q1–Q3: 34–52 days), compared to a delay of 35 days (Q1–Q3: 19–47 days) in group A (*p* = 0.004; Figure 1). Additionally, we observed a significantly higher pathological T classification in group B, when compared to group A (*p* = 0.046), while there was no difference in pathological N classification between groups (*p* = 0.843). There was a tendency of higher UICC stages in group B in comparison to group A, although the difference was not statistically significant (*p* = 0.116).

**TABLE 1:** Characteristics of the assessed patients with oral squamous cell carcinoma

Parameter	All patients 2010–2020, no. of patients	Group A 2010–2019, no. of patients	Group B 2020, no. of patients	*p*‐value
Total no. of patients per group	624	566	58	NA
Sex				
Female	258 (41%)	229 (40%)	29 (50%)	0.206 (chi‐square)
Male	366 (59%)	337 (60%)	29 (50%)	
Age				
Median	65	65	66	0.195 (tt2)
Q1–Q3	56–74	56–73	58–77	
Range	18–95	18–92	29–95	
Treatment delay				
Median	34	32	42	**0.004** (tt2)
Q1–Q3	20–48	19–47	34–52	
Pathologic tumor classification				
T1	218 (35%)	202 (36%)	16 (28%)	**0.046** (chi‐square)
T2	171 (27%)	160 (28%)	11 (19%)	
T3	51 (8%)	42 (7%)	9 (16%)	
T4	183 (29%)	162 (29%)	21 (36%)	
Tx	1 (1%)	NA	1 (1%)	
Pathologic nodal classification				
N0	401 (65%)	366 (65%)	35 (62%)	0.843 (chi‐square)
N1	53 (9%)	49 (9%)	4 (7%)	
N2a	5 (1%)	4 (1%)	1 (2%)	
N2b	38 (6%)	34 (6%)	4 (7%)	
N2c	21 (3%)	20 (4%)	1 (2%)	
N3a	0 (0%)	0 (0%)	0 (0%)	
N3b	98 (16%)	87 (16%)	11 (20%)	
Nx	8	6	2	
UICC stage				
Early stage (I/II)	287 (46%)	266 (47%)	21 (36%)	0.116 (chi‐square)
Advanced stage (III/IV)	337 (54%)	300 (53%)	37 (64%)	

*Note*: The values marked in bold are statistically significant (*p*‐values < 0.05). Abbreviations: chi‐square, Pearson's chi‐square test; tt2, Welch's two‐sample t test.

## 4 DISCUSSION

While we are continuously learning about the properties of Sars‐CoV‐2 and are developing better treatment strategies for COVID‐19, little is known about the impact that the pandemic has had on the therapy of patients with oral squamous cell carcinoma. In the present study, we demonstrated that the treatment delay in 2020 was prolonged compared to the reference period and that patients presented with more advanced tumor sizes.

Because the study necessarily demanded a retrospective design and because it was conducted at a single clinic, we cannot fully exclude the possibility of selection bias. Treatment delay may be dependent on the current severity of the pandemic and the occupancy rate of the health care system in the geographic area studied. Critically, the Heidelberg region had a rather moderate COVID‐19 incidence by national standards; therefore, more severely affected areas may face even greater treatment implications for non‐COVID patients. Thus, the effects we report here constitute conservative estimates and should be even more pronounced in more severely affected regions. The long‐term impacts that the observed treatment delay and larger tumor size will have still remain unknown and should be assessed conclusively in longitudinal studies. Because the study required a retrospective design, we were unable to examine the extent of patient delay. Furthermore, because of the relatively recent onset of COVID‐19, survival rates for both groups cannot be compared yet. Thus, the prognostic relevance of the reported treatment delays may only be estimated. Nonetheless, our data already demonstrate a significant and alarming impact of the COVID‐19 pandemic on the treatment schedules of patients suffering from oral cancer.

While treatment delay and tumor size are undoubtedly interlinked, we believe that treatment delay may not suffice to explain the reported differences in tumor sizes we find. Instead, an additional contributor might be a tendency that patients with oral lesions consulted later with a physician due to Sars‐CoV‐2‐related anxiety (i.e., patient delay). However, the retrospective study design precluded us from examining the extent of patient delay. Moreover, a potentially increased professional delay (i.e., time between presentation to a general practitioner and presentation to a specialist) may have led to further tumor growth. As hospital visits have been shown to carry a potential risk of Sars‐CoV‐2 infection, statements issued by the authorities in the past months have aimed at reducing the number of consultations.^5^

Indeed, new data from Turkey highlight a prolonged patient delay during the COVID‐19 pandemic with a high incidence of T3/T4 head and neck carcinomas and an increased mean time from onset of the first symptoms to hospital admission.^6^ In Japan, extended treatment delays have also been reported for patients with lung cancer during the COVID‐19 pandemic,^7^ while in China, an extended treatment delay for the initiation of radiotherapy in patients with nasopharyngeal carcinoma has been observed.^8^ In the context of the ongoing pandemic, other studies have reported a strong impact of COVID‐19 on patients with thoracic cancer, resulting in higher mortality and lower intensive care unit admissions.^9^

The impact of a prolonged treatment delay on survival rates has been discussed controversially and may be of varying relevance for different types of head and neck carcinomas.^10^, ^11^, ^12^ Recently published studies have shown that prolonged treatment delay negatively impacted the survival rates in patients with oral cavity cancer,^2^ especially in early‐stage disease,^13^ and have demonstrated that an increased treatment delay is an independent predictor of survival for patients with head and neck squamous cell carcinoma.^14^ This tendency is problematic when considering cancer patients with COVID‐19, because those patients were reported to be at a higher risk for severe illness which contributes even more to the risk of adverse outcomes.^15^ What is undisputed, however, is an increased morbidity and mortality associated with larger tumors, which require a comprehensive treatment regimen that includes both more extensive surgical and adjuvant procedures. Although the prognosis for patients with oral cancer has improved in recent decades,^16^ the COVID‐19‐related increases in tumor size have a significant impact on prognosis^17^ and quality of life.^18^, ^19^

Our recommendation is that if treatment delays occur due to limited resources, alternative treatment options should be presented, and advantages and disadvantages of these treatment plans must be weighed against the delay. It is noteworthy that head and neck surgeons continue to recommend primary surgical treatment for oral cavity cancers during the COVID‐19 pandemic.^20^ As far as is known, primary radiotherapy and radiochemotherapy are inferior to surgical treatment of early‐stage oral squamous cell carcinoma, whereas for only minor benefits for survival could be demonstrated for advanced‐stage disease.^21^ Nevertheless, the capacity of radiotherapy is also limited and carries other risks such as the occurrence of osteoradionecrosis.

Unfortunately, the trends toward delayed treatments and more advanced tumor sizes may continue in the next months and will possibly aggravate cancer treatment in the future. Therefore, physicians and responsible authorities should consider the presented results to prevent the deterioration of treatment outcomes.

## 5 CONCLUSION

While extraordinary efforts are continuously made to battle the COVID‐19 pandemic, it is imperative that this does not lead to significant disadvantages for many people with oral cancer. As treatment delay was significantly prolonged in 2020 compared to the reference period, and patients presented with more advanced tumor sizes, physicians and responsible authorities should consider the presented results to prevent a deterioration of patients' treatment during the ongoing COVID‐19 pandemic.

## Data Availability

Research data are not shared.
